# Backward Running: Acute Effects on Sprint Performance in Preadolescent Boys

**DOI:** 10.3390/sports8040055

**Published:** 2020-04-23

**Authors:** Dimitrios Petrakis, Eleni Bassa, Anastasia Papavasileiou, Anthi Xenofondos, Dimitrios A. Patikas

**Affiliations:** 1 Laboratory of Evaluation of Human Biological Performance, Faculty of Physical Education and Sport Science, Aristotle University of Thessaloniki, 54124 Thessaloniki, Greece; 2 Division of Kinesiology, School of Public Health, The University of Hong Kong, Pok Fu Lam 00000, Hong Kong

**Keywords:** preadolescence, child, post-activation performance enhancement, sprint, warm-up, rate of perceived exertion

## Abstract

The aim of this study was to examine the acute effect of backward running (BwR) during warm-up on a 20-m sprint of boys’ performance, compared to forward running (FwR). Fourteen recreationally active preadolescent boys (aged 12.5 ± 0.5 years) were examined in 3 protocols: warm-up (control condition), warm-up with 3 × 10 m additional BwR sprints and warm-up with 3 × 10 m additional FwR sprints. Participants were evaluated 4 minutes after each protocol on a 20-m sprint and intermediate distances, as well as the rate of perceived exertion (RPE). Sprint speed across 10-20 m was significantly higher for the BwR warm-up compared to the regular warm-up (*p* < 0.05) and a significantly higher RPE after the BwR and FwR protocols compared to the control condition was recorded (*p* < 0.05). No significant difference was detected across the distances 0–5, 5–10, 0–10 and 0–20 m. Although adding 3 × 10-m sprints of BwR or FwR after the warm-up did not enhance performance in a 20 m sprint of preadolescent boys, the positive effect of BwR across 10–20 m distance suggests that BwR could be an alternative means for enhancing performance for certain phases of a sprint for this age. However, preadolescent boys’ response to different sprint conditioning exercise stimuli and the optimization of rest time to maximize performance remain to be determined.

## 1. Introduction

Warm-up, as a common practice applied prior to exercise and sports activities, has the potential to improve performance [1]. There are several mechanisms that may contribute to this, such as increased muscle temperature [2,3], the elevation of oxygen uptake kinetics [4] and changes in the function of the neuromuscular system [5]. The inclusion of conditioning exercises in a warm-up, i.e. high-intensity exercises, is widely thought to potentiate performance [6,7]. “Post-activation performance enhancement” (PAPE) is a new term introduced by Cuenca-Fernández et al. [8] and describes such effects. In contrast to the classic post-activation potentiation, i.e. an increase in twitch force and power after electrically or voluntarily induced intense contraction [9,10], PAPE has a longer and weaker effect on performance, and is more likely attributed to different mechanisms [11]; the former is attributed to the phosphorylation of the myosin regulatory light-chain and the latter to changes in muscle temperature, muscle/cellular water content and/or muscle activation [11]. However, the acute effects of different conditioning stimuli—especially during warm-up—on the performance of tasks such as sprinting, is yet to be determined.

The effects of conditioning stimuli on sprint performance have been previously tested in adults. Effective PAPΕ effects have been reported using different types of conditioning stimulus, such as high resistance loads [6,12,13] or jumping exercises [14]. Although it was previously emphasized that PAPE stimulus is more effective when it is biomechanically similar to the subsequent activity [15], studies using sprints as conditioning stimuli to enhance sprint performance are limited [16,17]. These studies showed that adults did not improve their 60-m sprint speed after 2 × 60 m sprints [16], whereas young male track and field athletes increased their speed in a 100 m sprint after 2 × 20 m resisted sprints, and not after the same sprints as conditioning without resistance [17]. It seems therefore that the properties of the conditioning stimulus might be critical for the outcome of the study.

Regarding young ages and development, PAPE has not been extensively investigated in children prior to puberty. Although there are no differences in post-activation potentiation of the plantar flexor muscles between men, adolescents and pre-adolescents [18], it has been shown that after maximal half-squats, PAPE in terms of squat jump height was apparent in adult men but not in women, or adolescents and children of both sexes [19]. Similarly, in preadolescent female gymnasts, high-intensity task-specific (Rondat) or non-specific medium-intensity (double tuck jumps) conditioning contractions were not adequate to induce PAPE on drop jumps [20]. Nonetheless, there are indications that young adolescents can benefit from conditioning stimuli in the long-term. More specifically, resistance exercise can cause PAPE effects in adolescents only after and not before 10 weeks of resistance and sprint training [21]. Hence, it seems that the open question is not whether children are capable of demonstrating PAPE, but which are the optimal conditions and the appropriate candidates of conditioning stimuli to achieve it.

Running backwards (BwR) or forwards (FwR) are common types of movement in several sports [22,23,24], but there are several functional differences between them. Compared to FwR, BwR demonstrates greater lower limb muscle activation [25], higher rate of force development [26] and lower mechanical stress on the knee [27]. These properties suggest that BwR could be a promising, safe and efficient training stimulus. Furthermore, FwR and BwR differ in the type of contractions involved during the task. More specifically, BwR is associated more with concentric and less with eccentric work on the lower limbs [28]. This issue is of particular importance, because there is evidence that children, are not efficient in tasks that incorporate eccentric contractions, such as vertical jumps [29,30] and FwR [31], since they demonstrate prolonged contact time with the ground and hence inadequate transfer of energy among the joints. On the other hand, BwR is an effective training method to improve in the long-term children’s sprint speed [32], whilst there is no information regarding the acute effect of BwR on sprinting. To our knowledge, it is still unknown whether PAPE in pre-adolescent children’s sprinting performance could be induced by implementing BwR in a warm-up, i.e. a stimulus with a greater concentric contraction profile than FwR. Therefore, it remains to be tested if this effect of BwR would be greater than a warm-up protocol including FwR, which relies more on eccentric contractions. Considering the above, the aim of this study was to examine the acute effect of 3 × 10 m BwR bouts compared to FwR during a warm-up, on sprint performance, in pre-adolescent boys. We hypothesized that BwR would potentiate performance in a 20 m sprint and intermittent distances more than a typical warm-up program or a typical warm-up with FwR. This information could be useful for seeking methods to optimize sprint performance in children after their warm-up.

## 2. Materials and Methods

### 2.1. Participants

Fourteen (n = 14) recreationally active preadolescent boys (age: 12.5 ± 0.5 years; body mass: 50.2 ± 10.5 kg; height: 159.4 ± 10.1 cm) volunteered to participate in the study. This sample size for the present experimental design corresponds to 0.8 power, for 0.65 effect size at a = 0.05 (G-power, v.3.1.9.4, University of Kiel, Kiel, Germany). Maturity offset from peak height velocity was calculated according to the prediction equation based on anthropometric measures, sex and age [33] and the participants were characterized as pre-adolescents with a maturity offset of −2.11 ± 0.68 years. Body Mass Index (BMI) was calculated by the ratio of body mass to the body standing height squared (19.6 ± 2.8 kg/m^2^). All of them were healthy, with no musculoskeletal or neurological disease or lower limb injury. They joined two times a week for 90 min in a sports club, learning technical skills of team sports (soccer, handball, volleyball, basketball), in addition to the physical education class at school (according to school curriculum), two times per week for 45 min. Boys were asked to refrain from intense training 24 h prior to the testing days. Subjects’ parents/legal guardians were informed about the experimental process and signed informed consent for the participation of their son/legal ward. The study was conducted according to the Declaration of Helsinki and was approved by the institutional research review board (EC-1/2020).

### 2.2. Experimental Design/Procedures

A randomized controlled design was used to investigate the acute effect of three warm-up protocols on 20-m sprint performance in preadolescent boys. The intervention protocols consisted of (a) a typical warm-up (control: CON), (b) 3 × 10-m maximal BwR bouts in addition to the typical warm-up, and (c) 3 × 10-m maximal FwR bouts added to the typical warm-up. Each of these protocols was assessed in random order, at three sessions carried out on non-consecutive days, separated by 72 h, at an indoor gym (wood parquet flooring), at a regular time of the day (14:00–16:00) in order to minimize any possible impact of testing time [34]. Each protocol lasted approximately 8–9 min. The participants wore light clothing and the same footwear during each session. 

Rating of perceived exertion (RPE) was acquired immediately after the execution of each warm–up. The participants were tested in a 20-m sprint 4 min after the completion of each warm-up, in order to avoid fatigue [12,13,19]. Only one trial was performed since consecutive assessments could affect the performance of each subsequent sprint. The same investigator supervised all procedures and measurements.

One week before the first session, all participants were familiarized with the 20-m sprint and BwR [35]. Special attention was focused on the correct BwR technique, by means of demonstration and verbal feedback, following the guidelines of Uthoff et al. [32]. During the first session, anthropometric data of all participants were collected. A digital scale (BC-543, TANITA, Tokyo, Japan) and a stadiometer (Bodymeter 206, Seca, Ningbo, China) was used to measure body mass to the nearest 0.1 kg and body height (standing and seated) to the nearest 0.1 cm, respectively.

### 2.3. Intervention

The control condition of the typical warm–up (CON), lasted approximately 8 min and consisted of 3 min jogging at a low–medium tempo, followed by dynamic stretching exercises for the lower limbs (Table 1). More specifically, the first 7 exercises were performed for a 10-m distance and after the end of each exercise the participant walked back to the starting point. Dynamic stretching was preferred to static, to eliminate any potential adverse effect in performance [36]. 

The two other conditions consisted of three additional sets of 10 m maximal BwR or FwR sprints. Participants returned to the starting position running forward at a low pace. Subjects received verbal encouragement during BwR and FwR to ensure that the conditioning stimulus was maximal. 

### 2.4. 20 m Sprint Test

Sprint time over 5, 10 and 20 m was measured during the 20 m sprint (Table S1). For this purpose, three photocell timing gates (Witty Wireless Training Timer, Microgate, Bolzano, Italy) were placed at 5, 10, and 20 m. Photocells were adjusted to the pelvis height of each participant [37]. Participants were instructed to start after the verbal signal “ready, go”. They stood on an upright stride stance position, with their preferred foot forward, placed on the starting line over a pressure pad. Timing started when participants’ foot was detached from the pressure pad. The assessor ensured no false steps before starting and correct starting posture before the start. During the sprints verbal encouragement motivated for maximal effort. Sprint speed was analyzed for the distances 0–5, 5–10, 0–10, 10–20, and 0–20 m and was calculated by dividing the running distance by the time. 

### 2.5. Rate of Perceived Exertion (RPE)

RPE was measured immediately after the completion of each intervention (Table S1), using the 10-degree Children’s OMNI scale [38]. Participants replied to the question “*how tired do you feel?*”, while the investigator showed them the schematic OMNI scale. Participants had to declare the requisite exertion by indicating a number on the scale from 0 (not tired at all) to 10 (very, very tired). During the last two sessions at the sports club they were familiarized with the scale. This included a thorough description and explanation of the scale and responding to any questions or doubts that they had. 

### 2.6. Statistical Analysis

All data are presented as means and standard deviations. The dependent variables were the sprint speed for the distances of 0–5, 5–10, 0–10, 10–20, and 0–20 m and the RPE. The Shapiro-Wilk test was used to confirm the normal distribution of the data (*p*-values ranging from 0.126 to 0.850 among all variables), and Levene’s test for the equality of variances (*p* = 0.368–0.787). Furthermore, Mauchly’s test was performed to confirm that the assumption for sphericity was satisfied (*p* = 0.067–0.641). One-way Analysis of Variance (ANOVA) for repeated measurements was used for the statistical assessment to examine the effect of warm-up protocol (three levels: CON, BwR and FwR). The level of significance α was set at 0.05. Statistically significant effects were assessed with the Scheffé's post-hoc test. The effect sizes were calculated using eta squared (η^2^). The one sample t-test was used to examine the change in percent of sprint performance during the BwR or FwR relative to the CON condition compared to baseline zero. Confidence intervals at 95% confidence level (CI_95%_) were constructed. Statistical analysis was performed with SPSS for Windows, version 25 (IBM Corp., Armonk, NY, USA) and custom scripts in R, version 3.6.1 (R Foundation for Statistical Computing, Vienna, Austria).

## 3. Results

### 3.1. Sprint Speed

Sprint speed was not affected by protocol for the distances 0–5 m (F(2,26) = 0.34, *p* > 0.05, η^2^ = 0.03), 5–10 m (F(2,26) = 0.27, *p* > 0.05, η^2^ = 0.04), 0–10 m (F(2,26) = 0.46, *p* > 0.05, η^2^ = 0.10) and 0–20 m (F(2,26) = 0.79, *p* > 0.05, η^2^ = 0.06) (Table 2). However, a statistically significant effect of protocol on sprint speed was detected for the 10-20m distance (F(2,26) = 5.85, *p* = 0.008, η^2^ = 0.31). More specifically, post-hoc tests revealed significantly higher sprint speed over the 10-20m distance after the BwR protocol compared to control (*p* = 0.019, CI_95%_: 0.025 to 0.30).

**Figure 1:**
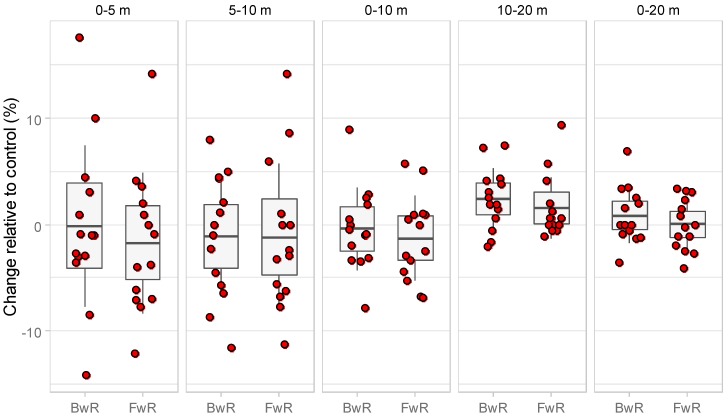
Percent change in 20 m sprint speed and its intermittent distances after the BwR and FwR protocols relative to the control protocol. Gray area corresponds to the CI_95%_ and vertical lines to the SD of mean, shown as a horizontal line in the middle of the gray area.

The percent change in sprint speed after the BwR and FwR relative to the CON protocol was highly variable among subjects for the distances 0–5 m, 5–10 m and 0–10 m, revealing participants with either lower or higher performance than the CON protocol (Figure 1). More systematic trends were observed for 10–20 m and 0–20 m distances. More specifically, for the distance 10–20 m the speed after the BwR protocol was 2.4 ± 2.9% higher than the CON and it was statistically different from zero (CI_95%_: 0.8 to 4.1% *p* = 0.008) while the increase by 1.6 ± 2.9% for the FwR compared to the CON was not significantly different from zero (CI_95%_: −0.1 to 3.3%, *p* = 0.065). Regarding the 0–20 m distance speed after the BwR was 0.9 ± 2.6% higher than the CON and 0.0 ± 2.4% after the FwR protocol. Both percentages were not significant from zero (BwR CI_95%_: −0.6 to 2.4 *p* = 0.241, and FwR CI_95%_: −1.4 to 1.4 *p* = 0.972, respectively).

### 3.2. Rate of Perceived Exertion

A statistically significant effect of protocol was found on RPE (F(2,26) = 24.2, *p* < 0.001). Post-hoc tests revealed a statistically significantly lower RPE in the CON protocol (1.9 ± 0.8, *p* < 0.001) compared to the BwR (4.1 ± 1.5) and FwR (4.2 ± 1.2) warm-up protocols. This indicates that implementing either 3 × 10 m BwR or FwR after a typical warm-up causes a similar RPE, which is higher relative to the typical warm-up per se.

## 4. Discussion

Adding 3 × 10 m sprints after a regular warm-up, regardless of the direction of running (BwR or FwR), caused a higher RPE but no significant improvement in the 20 m sprint speed compared to the typical warm-up (CON). Similarly, no significant effect of the warm-up protocol was observed for all intermittent distances of the sprint, except for the 10–20 m, where only the BwR protocol was superior compared to the CON in terms of sprint speed. Although the initial hypothesis for improved performance in 20 m sprint speed after the BwR compared to the FwR or CON protocols was not confirmed, these findings may give some limited evidence, that BwR could be an alternative means for enhancing performance for certain phases of a sprint in preadolescent boys.

To the best of our knowledge, this is the first study to examine the acute effect of running stimuli on sprint performance in preadolescent boys. Thus, it is difficult to directly compare the results of this study to those of other PAPE studies because of methodological differences. However, the fact that in the present study BwR or FwR failed to trigger a PAPE effect on 20-m sprinting performance in preadolescent boys is in accordance with previous research, regarding squat jumps with maximal isometric half-squats as conditioning [19]. More specifically, Arabatzi et al. [19], showed that among adults, adolescents and preadolescents of both sexes, jumping performance improved only in adult males and not in the other age and sex groups. One possible reason for the absence of PAPE in children could be their muscle fiber distribution [39], which is possibly lower in fast-twitch muscle fiber content, that are more prone to post-activation potentiation [40]. Furthermore, the training level seems to play a crucial role for the appearance of PAPE [21], and athletes with a high level of power or strength show a greater PAPE effect than athletes with lower values of power or strength [41,42]. More particularly, regarding sprinting speed, increased muscle stiffness and improved capacity to use effectively the stretch-shortening cycle are two factors linked to sprint performance and might be affected by training [43,44]. On the other hand, children have more compliant musculotendinous system [45,46], and insufficiently use their stretch-shortening cycle [29,30]. Recent studies indicate training may improve the former [32] but not the latter [47] in young athletes (adolescents and preadolescents, respectively). Hence, the existence of an immature neuromuscular system might also explain why the children that participated in the current study, which were in principle untrained (recreationally active), had no significant improvement in their 20 m sprint speed.

Beyond the age and training level, there are some additional factors that might have influenced the amount of PAPE on the 20-m sprint speed after the tested protocols. One of these factors could be the conditioning stimulus properties. The great variability of the effect of the conditioning stimulus, especially during the first 10 m, suggests that the optimal conditioning stimulus should be individualized as proposed by previous researchers [6,48]. This possibly explains the absence of differences in sprint speed between the protocol during the first 10 m. Previous studies have suggested that the reason for no positive effect of explosive conditioning stimuli on PAPE in 11- to 13-year-old gymnasts could be the relatively low volume and intensity [20]. In the present study, the effort of the trials could not be further increased since it was maximal. However, although there are possibilities to increase the load on the muscles, by adding resistance during the sprint, studies in adults have shown that performing sprint with resistance (backward sled towing) as conditioning did not improve their sprint speed for the first 5 m [49], which is in agreement with the current study. Nonetheless, the increased RPE observed after the end of the BwR and FwR protocol should be considered when planning future studies because in the presence of fatigue, adverse effects in performance might be expected [50]. Therefore, attempts to further increase the number of repetitions or the total covered distance, or the resistance during running (e.g., elastic bands), might have adverse effects on performance. However, the optimal load to maximize performance is still unknown.

Moreover, sprints require anaerobic power [51], whereas children have a decreased capacity to utilize their anaerobic metabolism [52,53]. Furthermore, sprinting, as a multi-joint, complex, circular and dynamic motion [54], is a challenging task for untrained children that have limited capacity to coordinate and activate optimally their muscles during complex movements [30,55]. In agreement with other studies [56,57,58], the lack of lower limb neuromuscular coordination might also explain the greater variability in percent change among the tested protocols, especially during the first 10 m of the acceleration, when the coordination demands are higher [51]. It is possible though that a larger sample size (n > 14) could reduce the probability for type II error in the case of 0–10 m distance. Alternatively, the lower variability shown at the 10–20 m distance could reveal a statistical differentiation in the BwR compared to the CON warm-up protocol. Hence, not only the volume and intensity, but also the nature of the conditioning stimulus could also play a role on the absence or presence of a PAPE effect. 

This was also the main purpose of the study, i.e. to evaluate the PAPE effect of two protocols with conditioning stimuli of different nature (BwR and FwR) compared to the CON condition. Indeed, for the distance 10–20 m, a warm-up including the BwR was superior in terms of sprint speed compared to the CON protocol (mean difference 0.16 m/s), whereas this was not the case for the FwR protocol. One explanation for this limited but statistically significant difference could be that BwR might be a better conditioning stimulus, since it involves more concentric contractions [28] and children are not able to execute eccentric movements involving the stretch-shortening cycle, as effectively as adults do [29,30]. However, considering that using eccentric contractions as conditioning stimulus is more effective than concentric [59], suggesting BwR as a means of inducing PAPE is still a compromise. Therefore, BwR could be suggested for novice athletes to improve their performance, but the main goal of the strength and conditioning trainer should be to improve their technique and performance using—among others—plyometric programs, which are effective in young ages [60,61]. 

Another factor that might contribute to the presence of PAPE, is the optimal timing between the end of the conditioning stimulus and the test [7,41]. Immediately after the end of the conditioning stimulus, fatigue may mask any PAPE effect [50]. The fact that in the present study the sprint speed after the BwR or FwR was not lower than the CON protocol, shows that despite the increased RPE values, a rest interval of 4 minutes after the conditioning was enough to maintain performance levels. Nonetheless, considering previous findings showing that children, compared to adults, recover faster, rely more on their aerobic mechanisms for energy production, and are more resistant to fatigue [53], it is reasonable to argue that shorter rest intervals might have the potential for greater PAPE in children. However, this requires further investigation.

Regarding RPE and metabolic cost, BwR at maximal intensity is considered to have greater energy consumption than FwR [26]. Nonetheless, in a recently published paper, RPE and metabolic cost during BwR and FwR, at self-pace speed, was similar [62]. Both of the previously mentioned studies involved adults. Considering the above, it could be assumed that one of the reasons why children had no significant difference in RPE between the BwR and FwR protocols in the present study, could be their potential inability to perform the task maximally. However, this assumption requires further investigation in the future to be verified.

From a practical point of view, the findings of this study support the inclusion of BwR sprints in warm-up routines in preadolescent children, as a method to improve sprint performance across 10–20 m distance. This acute effect in performance may enhance performance during training or competition. However, these findings regard recreationally active preadolescent children and cannot be generalized to the population of any specific sport. Each sport has different demands and the training stimuli may vary as well. Therefore, the existence and extent of improvement in sprinting velocity after BwR sprints, remains to be verified, for distances that are of specific interest to each sport. 

## 5. Conclusions

Although the implementation of 3 × 10 m sprints, either BwR or FwR, to a warm-up does not enhance 20 m sprint speed in recreationally active preadolescent boys, after a recovery period of 4 min, the positive effect of BwR on sprint speed during the distance 10–20 m suggests that BwR might be an alternative means for enhancing performance in certain phases of a sprint speed. However, preadolescent boys’ response to different sprint conditioning exercises, optimal rest time and/or conditioning stimuli remains to be determined on an individual basis, taking into account the basic characteristics and limitations of children’s physiology. 

## Figures and Tables

**Table 1:** The dynamic stretching exercises performed for 10 m after the 3 min jogging.

1. Hip in.	3. Heel kicks	5. Side steps (1 per side)	7. Knee hugs
2. Hip out	4. Speed skips	6. Karaoke (1 per side)	8. Front leg swings (10 per leg)

**Table 2:** Mean and standard deviation (SD) values of sprint speed (m/s) for the 20 m sprint and its intermittent distances for the warm-up protocols (CON: typical warm–up; BwR: typical warm–up plus 3 × 10 m backward running bouts; FwR: typical warm–up plus 3 × 10 m forward running bouts). Significantly higher values compared to the CON protocol are designated with asterisks (*: *p* < 0.01).

Distance	CON	BwR	FwR	*p*-Value
0–5 m	4.64 ± 0.28	4.66 ± 0.38	4.58 ± 0.34	0.714
5–10 m	5.53 ± 0.45	5.47 ± 0.29	5.47 ± 0.37	0.769
0–10 m	5.04 ± 0.27	5.02 ± 0.28	4.98 ± 0.29	0.634
10–20 m	6.15 ± 0.50	6.31 ± 0.61 *	6.25 ± 0.56	0.008
0–20 m	5.53 ± 0.33	5.58 ± 0.37	5.54 ± 0.38	0.465

## Supplementary Materials

The following are available online at https://www.mdpi.com/2075-4663/8/4/55/s1, Table S1: Speed and RPE Values of All Participants.
